# Molecular Epidemiology of Human Oral Chagas Disease Outbreaks in Colombia

**DOI:** 10.1371/journal.pntd.0002041

**Published:** 2013-02-21

**Authors:** Juan David Ramírez, Marleny Montilla, Zulma M. Cucunubá, Astrid Carolina Floréz, Pilar Zambrano, Felipe Guhl

**Affiliations:** 1 Centro de Investigaciones en Microbiología y Parasitología Tropical (CIMPAT), Universidad de Los Andes, Bogotá, Colombia; 2 Grupo de Parasitología, Instituto Nacional de Salud, Bogotá, Colombia; 3 Subdirección de Vigilancia y Control en Salud Pública, Instituto Nacional de Salud, Bogotá, Colombia; Universidad de Buenos Aires, Argentina

## Abstract

**Background:**

*Trypanosoma cruzi*, the causative agent of Chagas disease, displays significant genetic variability revealed by six Discrete Typing Units (TcI-TcVI). In this pathology, oral transmission represents an emerging epidemiological scenario where different outbreaks associated to food/beverages consumption have been reported in Argentina, Bolivia, Brazil, Ecuador and Venezuela. In Colombia, six human oral outbreaks have been reported corroborating the importance of this transmission route. Molecular epidemiology of oral outbreaks is barely known observing the incrimination of TcI, TcII, TcIV and TcV genotypes.

**Methodology and Principal Findings:**

High-throughput molecular characterization was conducted performing MLMT (Multilocus Microsatellite Typing) and mtMLST (mitochondrial Multilocus Sequence Typing) strategies on 50 clones from ten isolates. Results allowed observing the occurrence of TcI, TcIV and mixed infection of distinct TcI genotypes. Thus, a majority of specific mitochondrial haplotypes and allelic multilocus genotypes associated to the sylvatic cycle of transmission were detected in the dataset with the foreseen presence of mitochondrial haplotypes and allelic multilocus genotypes associated to the domestic cycle of transmission.

**Conclusions:**

These findings suggest the incrimination of sylvatic genotypes in the oral outbreaks occurred in Colombia. We observed patterns of super-infection and/or co-infection with a tailored association with the severe forms of myocarditis in the acute phase of the disease. The transmission dynamics of this infection route based on molecular epidemiology evidence was unraveled and the clinical and biological implications are discussed.

## Author Summary

Chagas disease represents a serious health problem affecting more than 10 million people in the Americas. The oral transmission route has emerged as a new epidemiological scenario that needs to be considered in prevention and control strategies. Herein was developed a high-resolution molecular characterization using mtMLST and MLMT tools in order to unravel the molecular epidemiology and transmission dynamics drivers in six well-characterized human oral outbreaks in Colombia. We observed the majority of clones typed as TcI and one clone as TcIV. The analysis of mitochondrial haplotypes allowed us to observe a high frequency of sylvatic haplotypes and a low proportion of domestic haplotypes. Likewise, a tailored allelic profile by each outbreak was observed. Our results suggest that sylvatic populations of *T. cruzi* are the causative agents of Chagas disease oral outbreaks and these findings should help to pursue new initiatives of control and prevention in those areas where domiciliated vectorial transmission has been interrupted.

## Introduction

Chagas disease caused by *Trypanosoma cruzi* is considered a zoonotic and neglected disease that represents an important public health problem in the Americas. This parasite is mainly transmitted by the faeces of triatomine insects and shows tremendous genetic variability reflected in at least six Discrete Typing Units (DTU's) classified as *T. cruzi* I – *T. cruzi* VI [1], [2]. Some authors have demonstrated intraspecific genetic diversity within *T. cruzi* I suggesting the existence of genotypes associated to the domestic (TcIa), peridomestic (TcIb) and sylvatic (TcId) transmission cycles [3]. Other routes of disease transmission, such as blood transfusion, vertical transmission, organ transplantation and accidental laboratory contamination are considered relevant routes [4]. *Trypanosoma cruzi* transmission by vertical vias and by transfusion of contaminated blood products has become the main mechanisms of transmission in non-endemic countries, including the USA and Spain [5], [6]. Currently, it is estimated that 7,694,500 people are infected with this parasite in South, Central American countries and Mexico where control initiatives are being conducted to interrupt *T. cruzi* transmission by triatomines and the transfusion of blood products [4].

Transmission via oral route is a relevant and emerging scenario of Chagas disease. This via displays a habitual character in the primitive and endemic cycle of the parasite occurring through consumption of contaminated food and/or beverages by dejection of triatomines faeces [4]. Also by ingestion of uncooked meat, food or beverages contaminated with urine or anal secretions of infected marsupials [7]. The emergence of Chagas disease from oral routes of transmission, especially in the Amazon Region are based on the consumption of food contaminated by the failure to adopt adequate hygiene practices in food handling and human establishment in sylvatic habitats, which increases the risks associated with the proximity of sylvatic vectors and reservoirs [7]. Oral outbreaks of Chagas disease were first reported in the Brazilian Amazon basin in 1965, followed by reports in Argentina, Bolivia, Colombia, Ecuador and in Venezuela where specific outbreaks have occurred in urban areas [8]. The previously mentioned cases of *T. cruzi* oral transmission resulted from consumption of sugarcane (*Saccharum spp.*) juice, açai (*Euterpe oleracea*) juice, Guava (*Psidium guajava*) juice and meat from hunting animals. The majority source of contamination has been attributed to *T. cruzi* infected insects that are macerated with the sugarcane or the fruits used for juice preparation [8]–[13]. All of these acute Chagas disease outbreaks associated with food/beverage consumption display severe clinical features in comparison with those of patients that have been infected with *T. cruzi* by other transmission routes [4], [11], [14].

**Figure 1:**
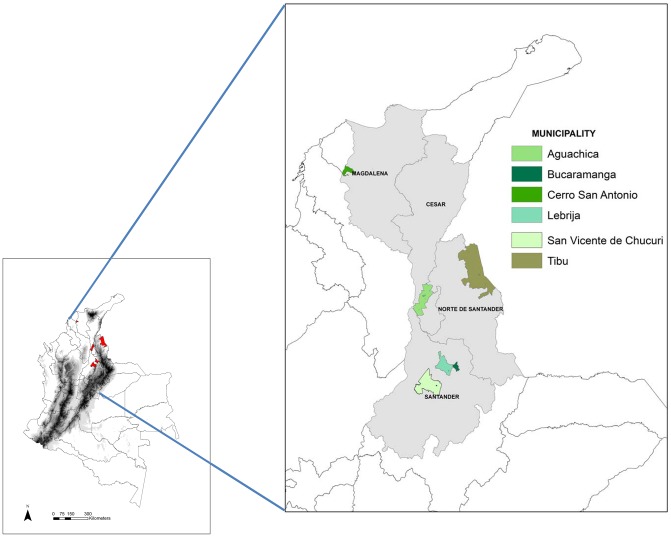
Geographical distribution and location of the municipalities in Colombia where oral transmission outbreaks have been reported.

In Colombia, six cases of acute Chagas disease outbreaks attributed to food/beverages consumption and catalogued as cases of oral transmission according to the National Health Institute (NHI) harboring four municipalities have been reported. The first outbreak occurred in a group of soldiers in the municipality of Tibú (Norte de Santander) in 1992. Six cases of acute Chagas disease myocarditis were confirmed. The NHI studied a group of 144 soldiers, of which 24 (17%) were confirmed as serologically positive by Immunofluorescent Assay (IFAT); Fifty-two percent of the seropositive patients presented electrocardiographic abnormalities [15]. The second outbreak was reported in the municipality of Guamal (Magdalena) in 1999. The surveys provided information from 607 patients; of these, 9 (3.2%) presented fevers. Sera samples were taken from 102 patients and 13% (13/102) of them were IFAT positive with cardiac alterations [16]. The third outbreak took place in Lebrija (Santander) in 2008, two patients died. Diagnosis was confirmed in 10 cases, which were 100% reactive to ELISA and IFAT. In addition, three cases presenting severe myocarditis were confirmed by histopathology [17]. The fourth outbreak took place in the city of Bucaramanga in 2009, when one child died due to severe myocarditis attributed to *T. cruzi* ethiology and a total of 5 cases were confirmed in the same family. The NHI found that the most frequent symptoms were fever (100%), abdominal pain (60%), and cardiomegaly (40%). Pericardial effusion was detected in 80% of the cases in the 2009 outbreak in Bucaramanga. The diagnosis was confirmed in 80% of the cases using serological tests (ELISA and IFAT). The two last outbreaks were reported in San Vicente de Chucurí (Santander) and Aguachica (Cesar) in 2010, when no deaths were reported but serological diagnosis confirmed that the symptoms were a result of Chagas disease. In the outbreak of Aguachica, 12 cases were confirmed by serological diagnosis using ELISA and IFAT; two cases presented pericardial effusion (Figure 1).

There is scarce available information regarding *T. cruzi* DTU's detected in the oral cases of Chagas disease. In studies developed by Marcili (2009), *T. cruzi* genotypes associated with food consumption were characterized from wild primates, triatomines and humans in the Brazilian Amazon; all isolates were genotyped as TcI and TcIV [18]. Andrade (2011) examined *T. cruzi* strains isolated from oral Chagas disease patients in Santa Catarina, Brazil and reported the presence of DTU's mixtures (TcI, TcII, TcV) [19]. Despite these efforts, there is not an evidence-based link between the severe clinical features of food-borne Chagas disease and *T. cruzi* genotypes [20]–[22]. The objective of this work was to develop a high-throughput analysis using polymorphic microsatellite markers based on a Multilocus Microsatellite Typing strategy (MLMT) and gene sequencing of the spliced leader intergenic region of mini-exon gene (SL-IR) and a mitochondrial Multilocus Sequence Typing (mtMLST) strategy to understand the molecular epidemiology of the clones isolated from six oral Chagas disease outbreaks in Colombia.

## Methods

### Study samples, isolates and clones

Eight *T. cruzi* isolates from humans (Tibú, Lebrija, Bucaramanga and San Vicente de Chucurí outbreaks) and two from triatomines (Guamal and Aguachica outbreaks) were cloned by a poisson-distributed limiting dilution assay, obtaining five clones from each isolate (a total of 50 clones analyzed) [23] (Table 1). Blood in guanidine buffer (GEB) from six patients (Lebrija outbreak) were collected and submitted to molecular characterization analysis by five genomic regions in order to discriminate *T. cruzi* DTU's [22], parasitic load quantification using a SYBR Green quantitative real-time PCR assay (qPCR), using methods previously reported [24] and Nested PCR of seven polymorphic microsatellite markers previously reported by following the amplification conditions described by Duque (2011) to determine the *T. cruzi* populations circulating in the patients [25], [26]. These microsatellite markers were only applied on this set of samples in terms of the sensitivity obtained since working with blood samples the number of parasites is scarce. An automated capillary sequencer (AB3730, Applied Biosystems, UK) was employed to obtain the allelic products using a fluorescent-tagged size standard. The metadata was checked manually for errors and all samples were typed in blind to avoid user bias.

**Table 1:** List of *Trypanosoma cruzi* stocks isolated from oral Chagas disease outbreaks in Colombia.

Isolate	Gender	Age (Years)	Outbreak	Year	Host	IFAT/ELISA	Cardiac alterations
MHOM/CO/92/FCH	Male	22	Tibú (Norte de Santander)	1992	*Homo sapiens*	NA	NA
IRHO/CO/99/SAN	-	-	Guamal (Magdalena)	1999	*Rhodnius pallescens*	+	+
MHOM/CO/09/LER	Male	21	Lebrija (Santander)	2008	*Homo sapiens*	+	+
MHOM/CO/09/EH	Male	22	Lebrija (Santander)	2008	*Homo sapiens*	+	+
MHOM/CO/09/XCH	Female	25	Bucaramanga (Santander)	2009	*Homo sapiens*	+	+
MHOM/CO/09/NCH	Male	48	Bucaramanga (Santander)	2009	*Homo sapiens*	+	+
MHOM/CO/09/LJVP	Female	22	Bucaramanga (Santander)	2009	*Homo sapiens*	+	+
MHOM/CO/10/SMA	Female	24	San Vicente de Chucurí (Santander)	2010	*Homo sapiens*	+	+
MHOM/CO/10/GC	Female	52	San Vicente de Chucurí (Santander)	2010	*Homo sapiens*	NA	NA
IRHO/CO/10/RPALL	-	-	Aguachica (Cesar)	2010	*R.pallescens*	+	+

Available sera samples from patients collected in the different outbreaks (four from Bucaramanga, two from San Vicente de Chucurí and four from Lebrija) were tested by TESA-blot. TESA-blot analysis was performed on sera previously positive by IFAT and ELISA. Briefly, TESAs from *T. cruzi* II strain Y were obtained from the supernatant of infected LLC-MK2 cells and used for immunoblotting. Membrane strips (5 mm) were later incubated with serum diluted 1∶200 in Tris-buffered saline (TBS)–1% milk for 2 h with mechanical agitation. After four 5-min washes in TBS, the bound antibodies were detected by using peroxidase-conjugated anti-human immunoglobulin G (Sigma) diluted 1∶2,500 in TBS–1% milk for 2 h. After a new cycle of washes, the immune complexes were revealed by the addition of H_2_O_2_ and 4-chloro-1-naphthol. The reaction was stopped with deionized water [27].

### Ethics statement

All of the appropriate ethical clearance was considered and approval of the ethics committee from Universidad de Los Andes under the form number 066/2006. Written consent was obtained in all human patients included as part of the epidemiological surveillance developed by NIH and Universidad de Los Andes under the same form number 066/2006.

### *T. cruzi* molecular characterization, microsatellite amplification and gene sequencing

Fifty clones corresponding to ten stocks were harvested until they reached logarithmic phase and 200 µL aliquots were taken for DNA extraction using the mini prep Qiagen kit (Table 1). We always used paired samples in order to compare DNA sequences and microsatellite allele profiles. The molecular characterization of the clones obtained was performed by amplifying the SL-IR and domain (D7) of the 24Sα region [28]. In order to detect genotypes within TcI, direct sequencing of SL-IR region and ten mitochondrion maxicircle DNA fragments was performed [28], [29]. Primers flanking variable regions of the pre-edited (12S rRNA and 9S rRNA), 5′ edited (Cytochrome b (Cytb), MUF1 (MurfA), NADH Dehydrogenase 1 (ND1), MURF2 (MurfB)), internal editing (Cytochrome oxidase II (COII)) and the non-edited regions, (NADH dehydrogenase 4 (ND4) and NADH dehydrogenase 5 (ND5a and ND5b), were amplified [29]. The amplification of the SL-IR and the ten maxicircle gene fragments was performed in a final volume of 20 µL using 1× Buffer (Corpogen, COL), 50 mM MgCl_2_, 10 µM of each primer, 5 U/µL of Taq Tucan (Corpogen, COL) and 10 ng of DNA. The mix was submitted to 29 cycles of amplification and the amplicons were visualized in 2% agarose gels stained with ethidium bromide. The PCR products were cleaned up by isopropanol precipitation and sequenced by the dideoxy-terminal method in an automated capillary sequencer (AB3730, Applied Biosystems, UK). The resulting sequences were edited in MEGA 5.0 [30]. All edited sequences were deposited in GenBank and assigned accession numbers KC282902–KC282951 (Table S1). The final sequences were concatenated in SeaView 4.0 according to the orientation of the kDNA maxicircle [31], [32]. The sequences of the maxicircle molecule were aligned and a matrix was constructed in Nexus format to develop a haplotype network analysis including reference strains from domestic and sylvatic mitochondrial haplotypes (EM – *Homo sapiens*-TcIa and YDm1M – *Didelphis marsupialis*-TcId), using the median-joining method and the default parameters on Network 2.0 to observe the polymorphism differences among the sequences. Twenty-four microsatellite loci were amplified as previously described [33]. These markers are spread over eight different chromosomes. An automated capillary sequencer (AB3730, Applied Biosystems, UK) was used to obtain the allelic products using a size standard with a fluorescent tag. The metadata was checked manually for errors and all samples were typed in blind to avoid user bias. Finally, individual level pair-wise distances were calculated using the DAS algorithm (1-proportion of shared alleles at all loci/n) and estimated using MICROSAT to construct a genetic distances Neighbor-joining tree (NJ).

## Results

### High-resolution molecular characterization of *T. cruzi* clones

**Figure 2:**
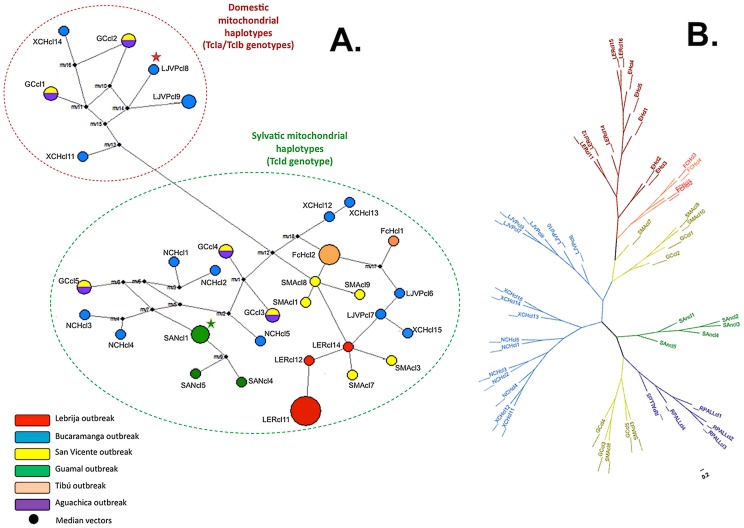
Network haplotype and un-rooted DAS tree of *T. cruzi* biological closes isolated from the six cases of oral Chagas disease transmission. **A.** Median-Joining haplotype network based on MLSTmt of 49 TcI clones isolated from oral outbreaks in Colombia shows the presence of 32 different haplotypes and the discrimination of domestic and sylvatic mitochondrial haplotypes according to the median vectors. Red star indicate the reference sequence of TcI domestic mithocondrial haplotype (EM) and previously typed as TcIa using SL-IR region. Green star indicate the reference sequence of TcI sylvatic mitochondrial haplotype and previously typed as TcId using SL-IR region. There is congruence between MLSTmt and SL-IR genotypes that convey in domestic haplotypes for TcIa/TcIb and sylvatic haplotypes for TcId. The black spots are considered ‘mv’ that can be biologically interpreted as possibly extant unsampled sequences or extinct ancestral sequences. **B.** Neighbor-Joining genetic distance tree based on MLMT of 49 TcI clones isolated from the oral outbreaks in Colombia shows the clustering of the allelic profiles within each outbreak. A significant number of allelic multilocus genotypes can be considered within each cluster. The topology of the un-rooted tree demonstrates the allelic relatedness among the clones isolated within the same oral outbreak with the presence of some outliers.

Molecular typing was performed in clones from the stocks, which resulted in the presence of *T. cruzi* I in 49 (98%) clones and *T. cruzi* IV in 1 (2%) clone (FCHcl5). When the sequencing of SL-IR was performed, occurrence of 11 (22%) clones as TcIa, 7 (14%) clones as TcIb and 31 (64%) clones as TcId was observed. According to the sequences of the ten gene fragments from the maxicircle molecule, a network was developed based on a median-joining algorithm, which allowed us to observe 32 different haplotypes among the dataset. From the network, we could establish six mitochondrial haplotypes associated to the domestic cycle and 24 mitochondrial haplotypes associated to the sylvatic cycle of transmission based on reference strains (TcIa and TcId), demonstrating the features displayed by each clone (Figure 2A). Based on the microsatellite patterns, a Neighbor-Joining tree was constructed using the genetic distances of shared alleles. We determined the genetic relationships between clones from the same oral outbreak, observing specific alleles groups in each oral outbreak (evidenced by the different number of multilocus genotypes), displaying that independent populations are the causative agents of each outbreak (Figure 2B; Table S1).

### Parasitic load and molecular characterization of GEB samples and TESA-blot results

The molecular characterization of GEB samples resulted in the detection of different genotypes within the same patient and absolute parasitic loads ranging from 110 to 250 parasites/mL (Table 2). In the cases of AM and EH, a co-infection with genotypes TcIa and TcId (SL-IR) was observed and also presenting the same allelic profile based on microsatellite data. Hence, patients JR and EB were found infected with the TcIa genotype and the same *T. cruzi* population. In the cases of patients FM and WM, they were infected with only the TcId genotype and they had the same allele profile based on microsatellite data. All of the samples analyzed by TESA-blot showed bands between 130–200 kDa, which corresponds to the SAPA antigen only detected in the acute phase of Chagas disease.

**Table 2:** DTU discrimination, TcI genotypes and polymorphic microsatellites (Allele sizes in base pair) results from GEB samples.

PATIENT	qPCR (parasites/mL)	DTU	TcI SL-IR genotype	TcAAAT6	TcAAT8	TcGAG10	TcCAA10	TcATT14	TcTAC15	TcTAT20
AM	125	TcI	TcIa-TcId	251/251	226/226	144/144	125/125	250/253	93/93	181/181
EH	255	TcI	TcIa-TcId	251/251	226/226	144/144	125/125	250/253	93/93	181/181
JR	130	TcI	TcIa	251/251	226/226	144/144	125/125	250/253	93/93	181/181
EB	110	TcI	TcIa	255/255	226/229	144/144	122/122	253/253	93/93	181/181
WM	250	TcI	TcId	251/255	226/229	144/144	122/125	250/253	93/93	181/181
FM	133	TcI	TcId	251/255	226/229	144/144	122/125	250/253	93/93	181/181

## Discussion

To our knowledge, this is the first report of a set of high-resolution molecular markers being applied to a group of clones isolated from cases of oral Chagas disease transmission. There are few reports where molecular discrimination of DTU's has been performed. In Brazil, there are reports of 14 isolates typed as TcI and TcIV in the Amazonian region [18]. In Santa Catarina, Brazil, a full study using several markers allowed the authors to find TcI, TcII, TcV and mixed infections of the isolates obtained from an oral outbreak in 2005 [19]. In Bolivia, a recent outbreak allowed the authors to find TcI and TcIV [34]. This suggests that our results showing a high prevalence of TcI and one clone typed as TcIV, are in accordance with the genotypes found in stocks isolated from other oral transmission cases. These findings are also in accordance with the biological and ecological distributions of TcI and TcIV, where these genotypes are related to the sylvatic transmission cycles and also display an interesting intersection feature being isolated from domestic and sylvatic hosts in a same niche [2], [33], [35], [36].

Information regarding the epidemiological surveillance was obtained in the cases of Lebrija, Bucaramanga and Aguachica. In Lebrija, the community collected two triatomines (*Panstrongylus geniculatus* and *Rhodnius pallescens*) from the sylvatic cycle where *T. cruzi* infection was detected in *P. geniculatus*. Regarding the reservoirs, one *D. marsupialis* was collected and not infected with trypanosomatids. As a result, we could determine those cases as oral transmission outbreaks due to the absence of domiciliated vectors. In Bucaramanga, the outbreak (nine cases) occurred in the same house during a citrus harvesting, in which the source of infection could be attributed to mandarine and/or orange juice. There was no evidence of domiciliated triatomines; *P. geniculatus* and *R. pallescens* were collected and *P. geniculatus* was found infected with *T. cruzi*. There are fruits that are sold in the area in little shops on the streets, where *P. geniculatus* vectors could infect the fruits by dejection of faeces, however further studies are required to validate this premise. The molecular characterization using SL-IR in the isolates and in the GEB samples showed that all clones from the Lebrija patients were typed as TcI. In addition, isolates from *P. geniculatus* have been found infected with TcI in the sylvatic foci, which may support that it could be the responsible vector in the outbreaks, including the similar multilocus genotypes evidenced by MLMT [23], [36]–[38]. These results are in accordance with the reported cases of acute oral Chagas disease in Venezuela, where *P. geniculatus* was the vector incriminated and TcI infection also confirmed [13], [39].

A significant frequency of TcI among the clones studied was found as previously reported in Colombia [28], [37]. In the samples collected, TcIa, TcIb and TcId were detected in the clones analyzed as mixed infections within stocks. Based on the analysis of SL-IR genotypes, a pattern of super-infection and/or co-infection within the same patient was observed. It is interesting to highlight the definition of super-infection in the acute phase of Chagas disease; in this context this might be suggesting that a composite of *T. cruzi* populations (from different triatomines and/or infected mammals) are causing the acute infection in the patients sampled. Hence, in terms of multiclonality the acute phase shows the lowest percentage of population diversity but this case is not ruled out in the oral transmission where at least two distinct genotypes were detected within the same patient. Therefore, the infection of two distinct genotypes was corroborated in the stocks LER and EH by microsatellites, demonstrating that certain alleles are associated to the domestic cycle of infection and some alleles associated with the sylvatic cycle residing within the same patient. This suggests that triatomines infected with a composite of *T. cruzi* populations are a source of infection or that the presence of more than one triatomine is involved in each outbreak. The TcIa genotype, which is associated with the domestic cycle of Chagas disease in Colombia, was detected but was not the most frequent. The TcIb genotype is associated with the peridomestic cycle, while TcId is associated with the sylvatic cycle and the most frequent in our dataset, which supports that the origin of infection was possible triatomines from sylvatic foci. The hypothesis about the origin of infection from sylvatic triatomines is supported by the presence of *P. geniculatus* and *R. pallescens* in the dwellings and because these triatomines were found to be mostly infected with genotypes TcIb and TcId [39]–[41].

A strong association across different haplotypes and the outbreaks was observed as well as the high genetic diversity among these clones represented in 32 different haplotypes, observing the occurrence of domestic and sylvatic haplotypes based on the reference strains sequences employed (Figure 2A) that correlates with the TcIa/TcIb (domestic genotypes) and TcId (sylvatic genotype) genotypes. The topology of the network suggests that the population affecting the two patients in Lebrija is similar and that the infection is harbored by a composite of *T. cruzi* populations, including a unique clone that has a hybrid maxicircle mosaic [37]. The network allows to observe the majority occurrence of sylvatic mitochondrial haplotypes and the low frequency of domestic mitochondrial haplotypes corroborating the premise of the likely incrimination of *T. cruzi* sylvatic DTU's in the oral infection outbreaks. *P. geniculatus* has been found naturally infected in Colombia with TcI, TcII and TcIII [23], [25], [42]. The presence of these DTU's in *P. geniculatus* is of paramount importance to understand the dynamics of *T. cruzi* DTUs in oral transmission outbreaks. The topology of the NJ tree based on polymorphic microsatellite data showed a strong association between each outbreak and the allelic profile of the clones obtained from each stock (Figure 2B). We did find defined clusters in most of the outbreaks with some outliers as seen in the San Vicente de Chucurí outbreak showing that the composite of *T. cruzi* populations causing the oral infection are quite alike and display a marked genetic variability pattern. There were two fatal cases in the Bucaramanga outbreak, which may suggest that the composition of multiclonal infections in oral outbreaks is closely related to the clinical manifestations of cardiomyopathy in acute cases of Chagas disease. Microsatellite markers were able to show the high multiclonality events observed in the stocks analyzed (a significant number of distinct allelic multilocus genotypes within hosts), which brings up the question whether the source of infection is the result of a single group of triatomines infected with the same *T. cruzi* population, or a composite of *T. cruzi* populations from the sylvatic cycle of transmission of Chagas disease in the region [35], [43]. The molecular characterization in GEB samples showed co-infections in two patients (AM and EH) with TcIa and TcId which supports the findings observed in the high-resolution molecular characterization performed in the clones from these patients (Table 2). In addition, the co-infection patterns could not be confirmed with the microsatellite markers employed. This premise emerges in the light of the absence of triple peaks among the loci studies or heterozygosis status; only locus TcATT14 showed heterozygosis and the rest of loci employed were homozygous, this could be explained by a low resolution power displayed by the microsatellite markers employed since parasite DNA yield was high (Table 2). Additionally, the multi-copy arrangement that SL-IR displays and the presence of null alleles and/or aneuploidy could explain these premises but further testing is required [44].

In conclusion, we developed a high-resolution molecular typing of the stocks isolated from cases of Chagas disease transmitted by an oral infection route in Colombia. We determined that the prevalent DTU in the stocks was TcI, with one clone typed as TcIV. Based on our results, the *T. cruzi* populations causing acute infections are those associated with the sylvatic foci and vectored by *P. geniculatus and R. pallescens*. The DTU discrimination in the oral infection outbreaks allowed us to highlight the significant impact of the DTU's from the sylvatic cycle, TcI and TcIV, in the outbreaks studied, which implies that oral transmission is a relevant epidemiological scenario that has emerged in the natural life cycle of *T. cruzi*. This suggests that, in areas where vectorial transmission has been interrupted, new acute cases of Chagas disease may emerge as a potential problem in public health.

## Supporting Information

Table S1 **Microsatellite alleles, SL-IR genotypes and accession numbers of the clones analyzed.**
(DOC)
