# Supplementary material for: Molecular Epidemiology of Human Oral Chagas Disease Outbreaks in Colombia
Source: PLoS Negl Trop Dis. 2013 Feb 21;7(2):e2041. doi: 10.1371/journal.pntd.0002041 (PMC3578743; doi:10.1371/journal.pntd.0002041)
Supplement: Table S1 — Microsatellite alleles, SL-IR genotypes and accession numbers of the clones analyzed. (DOC) [file pntd.0002041.s001.doc]

**Table S1. Microsatellite alleles, SL-IR genotypes and accession numbers of the clones analyzed.**

| **Clone** | **Accession number** | **SL-IR genotype** | **10101 CA a NED** | **10101 TA VIC** | **10101 TC VIC** | **10187 CA TA VIC** |
| --- | --- | --- | --- | --- | --- | --- |
| FCHcl1 | KC282902 | TcIb | 170/170 | 155/159 | 104/104 | 253/263 |
| FCHcl2 | KC282903 | TcIb | 170/170 | 155/115 | 104/104 | 261/261 |
| FCHcl3 | KC282904 | TcIb | 170/170 | 155/159 | 104/104 | 281/281 |
| FCHcl4 | KC282905 | TcIa | 170/170 | 155/159 | 104/104 | 281/281 |
| FCHcl5 | KC282906 | TcIV |  |  |  |  |
| SANcl1 | KC282907 | TcIb | 172/172 | 163/163 | 104/104 | 255/255 |
| SANcl2 | KC282908 | TcIb | 172/172 | 163/163 | 104/104 | 255/265 |
| SANcl3 | KC282909 | TcIb | 172/172 | 163/163 | 104/104 | 255/265 |
| SANcl4 | KC282910 | TcIb | 172/172 | 163/163 | 104/104 | 255/265 |
| SANcl5 | KC282911 | TcIb | 172/172 | 163/163 | 104/104 | 255/265 |
| LERcl11 | KC282912 | TcIa | 172/178 | 155/159 | 104/104 | 281/281 |
| LERcl12 | KC282913 | TcIb | 172/178 | 159/160 | 104/108 | 281/281 |
| LERcl14 | KC282914 | TcIa | 172/178 | 159/159 | 104/108 | 265/265 |
| LERcl15 | KC282915 | TcId | 172/178 | 159/159 | 104/108 | 265/265 |
| LERcl16 | KC282916 | TcId | 172/178 | 159/159 | 104/108 | 265/265 |
| EHcl1 | KC282917 | TcIa | 172/178 | 159/159 | 104/108 | 265/265 |
| EHcl2 | KC282918 | TcIa | 170/170 | 155/159 | 104/108 | 265/265 |
| EHcl3 | KC282919 | TcId | 172/178 | 155/159 | 104/104 | 265/265 |
| EHcl4 | KC282920 | TcId | 172/178 | 159/159 | 104/108 | 265/265 |
| EHcl5 | KC282921 | TcId | 172/178 | 159/159 | 104/108 | 265/265 |
| XCHcl11 | KC282922 | TcIa | 172/173 | 159/163 | 104/104 | 277/277 |
| XCHcl12 | KC282923 | TcIb | 172/172 | 159/163 | 104/104 | 277/277 |
| XCHcl13 | KC282924 | TcIa | 172/172 | 159/163 | 104/104 | 277/277 |
| XCHcl14 | KC282925 | TcIa | 172/172 | 159/163 | 104/104 | 277/277 |
| XCHcl15 | KC282926 | TcIa | 172/172 | 159/163 | 104/104 | 277/277 |
| NCHcl1 | KC282927 | TcIa | 172/172 | 159/163 | 104/104 | 277/277 |
| NCHcl2 | KC282928 | TcIa | 172/172 | 159/163 | 104/104 | 277/277 |
| NCHcl3 | KC282929 | TcIa | 172/172 | 159/163 | 104/104 | 277/277 |
| NCHcl4 | KC282930 | TcIa | 172/172 | 159/159 | 104/104 | 277/277 |
| NCHcl5 | KC282931 | TcIb | 172/172 | 159/163 | 104/104 | 277/277 |
| LJVPcl6 | KC282932 | TcId | 172/172 | 155/159 | 104/104 | 257/263 |
| LJVPcl7 | KC282933 | TcId | 172/172 | 159/159 | 104/104 | 257/263 |
| LJVPcl8 | KC282934 | TcIa | 172/172 | 159/159 | 104/104 | 257/263 |
| LJVPcl9 | KC282935 | TcIa | 172/172 | 159/159 | 104/104 | 257/263 |
| LJVPcl10 | KC282936 | TcIa | 172/172 | 159/159 | 104/104 | 257/263 |
| SMAcl3 | KC282937 | TcId | 172/172 | 159/164 | 106/106 | 257/257 |
| SMAcl7 | KC282938 | TcId | 172/180 | 155/159 | 104/104 | 257/263 |
| SMAcl8 | KC282939 | TcIb | 172/172 | 159/164 | 106/106 | 257/257 |
| SMAcl9 | KC282940 | TcIa | 172/172 | 155/159 | 104/106 | 257/263 |
| SMAcl10 | KC282941 | TcIa | 172/172 | 155/159 | 104/106 | 257/263 |
| GCcl1 | KC282942 | TcIa | 172/172 | 155/159 | 104/106 | 257/263 |
| GCcl2 | KC282943 | TcIa | 172/172 | 155/159 | 104/106 | 257/263 |
| GCcl3 | KC282944 | TcIb | 172/172 | 159/164 | 106/106 | 257/257 |
| GCcl4 | KC282945 | TcIb | 172/172 | 159/164 | 106/106 | 257/257 |
| GCcl5 | KC282946 | TcId | 172/172 | 159/164 | 106/106 | 257/257 |
| DScl1 | KC282947 | TcIa | 172/172 | 159/159 | 104/104 | 289/289 |
| DScl2 | KC282948 | TcIb | 172/172 | 159/159 | 104/104 | 289/289 |
| DScl3 | KC282949 | TcId | 172/174 | 159/159 | 104/104 | 289/289 |
| DScl4 | KC282950 | TcId | 172/172 | 159/159 | 104/104 | 289/289 |
| DScl5 | KC282951 | TcId | 172/172 | 159/159 | 104/104 | 289/289 |
| RPALLcl1 | KC282952 | TcIb | 172/172 | 159/164 | 106/107 | 257/257 |
| RPALLcl2 | KC282953 | TcIb | 172/172 | 159/164 | 106/106 | 257/257 |
| RPALLcl3 | KC282954 | TcIb | 172/172 | 159/164 | 106/106 | 257/257 |
| RPALLcl4 | KC282955 | TcId | 172/172 | 159/159 | 106/106 | 257/257 |
| RPALLcl5 | KC282956 | TcId | 172/172 | 159/159 | 104/108 | 257/257 |

| **Clone** | **SL-IR genotype** | **10359 CA NED** | **11283 TAb NED** | **11283 TCG FAM** | **11863 CA VIC** |
| --- | --- | --- | --- | --- | --- |
| FCHcl1 | TcIb | 137/141 | 169/169 | 138/138 | 116/116 |
| FCHcl2 | TcIb | 137/137 | 169/169 | 138/138 | 116/116 |
| FCHcl3 | TcIb | 137/141 | 169/169 | 138/138 | 116/116 |
| FCHcl4 | TcIa | 137/141 | 169/169 | 138/138 | 116/116 |
| FCHcl5 | TcIV |  |  |  |  |
| SANcl1 | TcIb | 139/155 | 166/174 | 132/132 | 116/116 |
| SANcl2 | TcIb | 139/153 | 166/174 | 132/132 | 116/116 |
| SANcl3 | TcIb | 139/153 | 166/174 | 132/132 | 116/116 |
| SANcl4 | TcIb | 139/153 | 166/174 | 132/132 | 116/116 |
| SANcl5 | TcIb | 139/153 | 166/174 | 132/132 | 116/116 |
| LERcl11 | TcIa | 145/149 | 169/174 | 138/138 | 115/117 |
| LERcl12 | TcIb | 141/151 | 172/172 | 126/138 | 116/116 |
| LERcl14 | TcIa | 141/151 | 172/172 | 126/138 | 116/116 |
| LERcl15 | TcId | 141/151 | 172/172 | 126/138 | 116/116 |
| LERcl16 | TcId | 141/151 | 172/172 | 126/138 | 116/116 |
| EHcl1 | TcIa | 141/151 | 172/172 | 126/138 | 116/116 |
| EHcl2 | TcIa | 137/141 | 169/169 | 138/138 | 116/116 |
| EHcl3 | TcId | 137/141 | 169/169 | 138/138 | 116/116 |
| EHcl4 | TcId | 141/151 | 172/172 | 126/138 | 116/116 |
| EHcl5 | TcId | 141/151 | 172/172 | 126/138 | 116/116 |
| XCHcl11 | TcIa | 141/162 | 169/169 | 126/135 | 116/116 |
| XCHcl12 | TcIb | 141/162 | 158/169 | 126/135 | 116/116 |
| XCHcl13 | TcIa | 141/162 | 169/169 | 126/135 | 116/116 |
| XCHcl14 | TcIa | 141/162 | 169/169 | 126/135 | 116/116 |
| XCHcl15 | TcIa | 141/162 | 169/169 | 126/135 | 116/116 |
| NCHcl1 | TcIa | 141/162 | 169/169 | 126/135 | 116/116 |
| NCHcl2 | TcIa | 141/162 | 169/169 | 126/135 | 116/116 |
| NCHcl3 | TcIa | 141/162 | 169/169 | 126/135 | 116/116 |
| NCHcl4 | TcIa | 141/162 | 169/169 | 126/135 | 116/116 |
| NCHcl5 | TcIb | 141/162 | 169/169 | 126/135 | 116/116 |
| LJVPcl6 | TcId | 141/160 | 169/169 | 126/126 | 116/116 |
| LJVPcl7 | TcId | 141/160 | 158/169 | 126/135 | 116/116 |
| LJVPcl8 | TcIa | 141/160 | 169/169 | 126/135 | 116/116 |
| LJVPcl9 | TcIa | 141/160 | 158/169 | 126/135 | 116/116 |
| LJVPcl10 | TcIa | 141/160 | 169/169 | 126/135 | 116/116 |
| SMAcl3 | TcId | 145/149 | 169/174 | 126/141 | 116/116 |
| SMAcl7 | TcId | 145/149 | 169/169 | 126/138 | 116/116 |
| SMAcl8 | TcIb | 145/149 | 169/174 | 126/141 | 116/116 |
| SMAcl9 | TcIa | 139/141 | 169/169 | 135/135 | 116/116 |
| SMAcl10 | TcIa | 139/141 | 169/169 | 135/135 | 116/116 |
| GCcl1 | TcIa | 139/141 | 169/169 | 135/135 | 116/116 |
| GCcl2 | TcIa | 139/141 | 169/169 | 135/135 | 116/116 |
| GCcl3 | TcIb | 145/149 | 169/174 | 126/141 | 116/116 |
| GCcl4 | TcIb | 145/149 | 169/174 | 126/141 | 116/116 |
| GCcl5 | TcId | 145/149 | 169/174 | 126/141 | 116/116 |
| DScl1 | TcIa | 145/149 | 169/169 | 126/126 | 116/116 |
| DScl2 | TcIb | 145/149 | 169/169 | 126/126 | 116/116 |
| DScl3 | TcId | 145/149 | 169/169 | 126/126 | 116/116 |
| DScl4 | TcId | 145/149 | 169/169 | 126/126 | 116/116 |
| DScl5 | TcId | 145/149 | 169/169 | 126/126 | 116/116 |
| RPALLcl1 | TcIb | 131/131 | 145/149 | 169/174 | 126/141 |
| RPALLcl2 | TcIb | 131/131 | 145/149 | 169/174 | 126/141 |
| RPALLcl3 | TcIb | 131/131 | 145/149 | 169/174 | 126/141 |
| RPALLcl4 | TcId | 131/131 | 145/149 | 169/174 | 126/141 |
| RPALLcl5 | TcId | 131/131 | 145/149 | 169/174 | 135/135 |

| **Clone** | **SL-IR genotype** | **6529 TA b PET** | **6559 TC TET** | **6855 TA GA NED** | **6925 CT VIC** |
| --- | --- | --- | --- | --- | --- |
| FCHcl1 | TcIb | 169/169 | 110/110 | 147/147 | 168/172 |
| FCHcl2 | TcIb | 169/172 | 110/110 | 147/111 | 168/168 |
| FCHcl3 | TcIb | 169/169 | 110/110 | 147/147 | 168/172 |
| FCHcl4 | TcIa | 169/169 | 110/110 | 147/147 | 168/172 |
| FCHcl5 | TcIV |  |  |  |  |
| SANcl1 | TcIb | 169/169 | 110/110 | 136/136 | 174/174 |
| SANcl2 | TcIb | 169/169 | 110/110 | 136/136 | 174/174 |
| SANcl3 | TcIb | 169/169 | 110/110 | 136/136 | 174/174 |
| SANcl4 | TcIb | 169/169 | 110/110 | 136/136 | 174/174 |
| SANcl5 | TcIb | 169/169 | 110/110 | 136/136 | 174/174 |
| LERcl11 | TcIa | 169/169 | 110/110 | 147/147 | 174/174 |
| LERcl12 | TcIb | 169/169 | 110/110 | 147/147 | 174/174 |
| LERcl14 | TcIa | 169/171 | 110/110 | 147/147 | 174/174 |
| LERcl15 | TcId | 169/171 | 110/110 | 147/147 | 174/174 |
| LERcl16 | TcId | 169/171 | 110/110 | 147/147 | 174/174 |
| EHcl1 | TcIa | 169/169 | 110/110 | 147/147 | 174/174 |
| EHcl2 | TcIa | 169/169 | 110/110 | 147/147 | 168/172 |
| EHcl3 | TcId | 169/169 | 110/110 | 147/147 | 178/172 |
| EHcl4 | TcId | 169/171 | 110/110 | 147/147 | 174/174 |
| EHcl5 | TcId | 169/171 | 110/110 | 147/147 | 174/174 |
| XCHcl11 | TcIa | 169/169 | 110/110 | 149/156 | 172/174 |
| XCHcl12 | TcIb | 169/169 | 110/110 | 149/156 | 172/174 |
| XCHcl13 | TcIa | 169/169 | 110/110 | 149/156 | 172/174 |
| XCHcl14 | TcIa | 169/169 | 110/110 | 149/156 | 172/174 |
| XCHcl15 | TcIa | 169/169 | 110/110 | 149/156 | 172/174 |
| NCHcl1 | TcIa | 169/169 | 110/110 | 149/156 | 172/174 |
| NCHcl2 | TcIa | 169/169 | 110/110 | 149/156 | 172/174 |
| NCHcl3 | TcIa | 169/169 | 110/110 | 149/156 | 172/174 |
| NCHcl4 | TcIa | 169/169 | 110/110 | 149/156 | 172/174 |
| NCHcl5 | TcIb | 169/169 | 110/110 | 149/156 | 172/174 |
| LJVPcl6 | TcId | 169/171 | 110/110 | 147/156 | 174/174 |
| LJVPcl7 | TcId | 169/171 | 110/110 | 147/156 | 172/174 |
| LJVPcl8 | TcIa | 169/171 | 110/110 | 147/156 | 172/174 |
| LJVPcl9 | TcIa | 169/171 | 110/110 | 147/156 | 172/174 |
| LJVPcl10 | TcIa | 169/171 | 110/110 | 147/156 | 172/174 |
| SMAcl3 | TcId | 169/169 | 108/110 | 149/152 | 172/174 |
| SMAcl7 | TcId | 169/169 | 110/110 | 147/152 | 172/172 |
| SMAcl8 | TcIb | 169/169 | 108/110 | 147/149 | 172/174 |
| SMAcl9 | TcIa | 169/171 | 110/110 | 147/152 | 172/172 |
| SMAcl10 | TcIa | 169/171 | 110/110 | 147/152 | 172/172 |
| GCcl1 | TcIa | 169/171 | 110/110 | 147/152 | 172/172 |
| GCcl2 | TcIa | 169/171 | 110/110 | 147/152 | 172/172 |
| GCcl3 | TcIb | 169/169 | 108/110 | 147/149 | 172/174 |
| GCcl4 | TcIb | 169/169 | 108/110 | 147/149 | 172/174 |
| GCcl5 | TcId | 169/169 | 108/110 | 149/152 | 172/174 |
| DScl1 | TcIa | 169/169 | 110/110 | 149/149 | 174/174 |
| DScl2 | TcIb | 169/169 | 110/110 | 149/149 | 174/174 |
| DScl3 | TcId | 169/169 | 110/110 | 149/149 | 174/174 |
| DScl4 | TcId | 169/169 | 110/110 | 149/149 | 174/174 |
| DScl5 | TcId | 169/169 | 110/110 | 149/149 | 174/174 |
| RPALLcl1 | TcIb | 169/169 | 108/110 | 149/152 | 172/174 |
| RPALLcl2 | TcIb | 169/169 | 108/110 | 149/152 | 172/174 |
| RPALLcl3 | TcIb | 169/169 | 108/110 | 149/152 | 172/174 |
| RPALLcl4 | TcId | 169/169 | 110/110 | 149/152 | 172/174 |
| RPALLcl5 | TcId | 169/169 | 110/110 | 149/152 | 168/168 |

| **Clone** | **SL-IR genotype** | **6925 TG a NED** | **6925 TGb NED** | **7093 TA b NED** | **7093 TA c TET** | **mclf10 FAM** | **TcUn3 PET** |
| --- | --- | --- | --- | --- | --- | --- | --- |
| FCHcl1 | TcIb | 142/142 | 109/111 | 112/112 | 186/190 | 185/185 | 146/146 |
| FCHcl2 | TcIb | 142/190 | 109/109 | 112/112 | 186/186 | 185/185 | 146/146 |
| FCHcl3 | TcIb | 142/142 | 109/111 | 112/112 | 186/190 | 185/185 | 154/154 |
| FCHcl4 | TcIa | 142/142 | 109/111 | 112/112 | 186/190 | 185/185 | 146/146 |
| FCHcl5 | TcIV |  |  |  |  |  |  |
| SANcl1 | TcIb | 140/140 | 111/111 | 109/112 | 182/182 | 185/188 | 154/156 |
| SANcl2 | TcIb | 140/140 | 111/111 | 109/109 | 182/182 | 185/185 | 154/156 |
| SANcl3 | TcIb | 140/140 | 111/111 | 109/112 | 182/182 | 185/188 | 154/156 |
| SANcl4 | TcIb | 140/140 | 111/111 | 109/112 | 182/182 | 185/188 | 154/156 |
| SANcl5 | TcIb | 140/140 | 111/111 | 109/112 | 182/182 | 185/188 | 154/156 |
| LERcl11 | TcIa | 145/149 | 109/111 | 107/107 | 188/188 | 179/179 | 154/158 |
| LERcl12 | TcIb | 140/140 | 109/109 | 112/112 | 188/188 | 179/179 | 154/158 |
| LERcl14 | TcIa | 140/140 | 109/109 | 112/112 | 188/188 | 182/185 | 146/152 |
| LERcl15 | TcId | 140/140 | 109/111 | 109/112 | 188/188 | 182/185 | 146/152 |
| LERcl16 | TcId | 140/140 | 109/111 | 109/112 | 188/188 | 182/185 | 146/152 |
| EHcl1 | TcIa | 140/140 | 109/111 | 109/112 | 188/188 | 182/185 | 146/152 |
| EHcl2 | TcIa | 140/142 | 109/111 | 112/116 | 186/188 | 185/185 | 146/146 |
| EHcl3 | TcId | 142/142 | 109/111 | 112/116 | 188/188 | 185/185 | 146/146 |
| EHcl4 | TcId | 140/140 | 109/111 | 109/112 | 188/188 | 182/185 | 146/152 |
| EHcl5 | TcId | 140/140 | 109/111 | 112/112 | 188/188 | 182/185 | 146/152 |
| XCHcl11 | TcIa | 140/140 | 109/109 | 112/116 | 184/186 | 185/185 | 146/146 |
| XCHcl12 | TcIb | 140/140 | 109/109 | 103/116 | 184/186 | 185/185 | 146/146 |
| XCHcl13 | TcIa | 140/140 | 109/109 | 112/112 | 184/186 | 185/185 | 146/146 |
| XCHcl14 | TcIa | 140/140 | 109/109 | 112/112 | 184/186 | 185/185 | 146/146 |
| XCHcl15 | TcIa | 140/140 | 109/109 | 112/112 | 184/186 | 185/185 | 146/146 |
| NCHcl1 | TcIa | 140/140 | 109/109 | 109/112 | 184/186 | 185/185 | 146/146 |
| NCHcl2 | TcIa | 140/140 | 109/109 | 103/112 | 184/186 | 185/185 | 146/146 |
| NCHcl3 | TcIa | 140/140 | 109/109 | 103/112 | 184/186 | 185/185 | 146/146 |
| NCHcl4 | TcIa | 140/140 | 109/109 | 112/116 | 184/186 | 185/185 | 146/146 |
| NCHcl5 | TcIb | 140/140 | 109/109 | 109/112 | 184/186 | 185/185 | 146/146 |
| LJVPcl6 | TcId | 140/140 | 109/109 | 103/112 | 188/188 | 185/185 | 146/146 |
| LJVPcl7 | TcId | 140/140 | 109/109 | 103/112 | 184/186 | 185/185 | 146/146 |
| LJVPcl8 | TcIa | 140/140 | 109/109 | 103/112 | 184/186 | 185/185 | 146/146 |
| LJVPcl9 | TcIa | 140/140 | 109/109 | 103/112 | 184/186 | 185/185 | 146/146 |
| LJVPcl10 | TcIa | 140/140 | 109/109 | 103/112 | 184/186 | 185/185 | 146/146 |
| SMAcl3 | TcId | 142/142 | 109/113 | 103/112 | 186/188 | 183/185 | 146/154 |
| SMAcl7 | TcId | 140/142 | 109/109 | 103/112 | 186/188 | 183/185 | 146/146 |
| SMAcl8 | TcIb | 142/142 | 109/113 | 112/116 | 186/188 | 183/185 | 146/154 |
| SMAcl9 | TcIa | 140/142 | 109/109 | 112/116 | 184/186 | 183/185 | 146/146 |
| SMAcl10 | TcIa | 140/142 | 109/109 | 112/116 | 184/186 | 183/185 | 146/146 |
| GCcl1 | TcIa | 140/142 | 109/109 | 112/116 | 184/186 | 183/185 | 146/146 |
| GCcl2 | TcIa | 140/142 | 109/109 | 112/116 | 184/186 | 183/185 | 146/146 |
| GCcl3 | TcIb | 142/142 | 109/113 | 112/116 | 186/188 | 183/185 | 146/154 |
| GCcl4 | TcIb | 142/142 | 109/113 | 112/116 | 186/188 | 183/185 | 146/154 |
| GCcl5 | TcId | 142/142 | 109/113 | 103/112 | 186/188 | 183/185 | 146/154 |
| DScl1 | TcIa | 140/140 | 109/109 | 116/116 | 188/188 | 183/185 | 154/154 |
| DScl2 | TcIb | 140/140 | 109/109 | 116/116 | 184/184 | 183/185 | 158/158 |
| DScl3 | TcId | 140/140 | 109/109 | 116/116 | 188/188 | 183/185 | 154/154 |
| DScl4 | TcId | 140/140 | 109/109 | 112/116 | 188/188 | 183/185 | 154/154 |
| DScl5 | TcId | 140/140 | 109/109 | 103/112 | 188/188 | 183/185 | 162/162 |
| RPALLcl1 | TcIb | 142/142 | 113/113 | 103/112 | 196/196 | 183/185 | 154/156 |
| RPALLcl2 | TcIb | 142/142 | 113/113 | 103/116 | 196/196 | 183/185 | 154/156 |
| RPALLcl3 | TcIb | 142/142 | 113/113 | 103/116 | 196/196 | 183/185 | 154/156 |
| RPALLcl4 | TcId | 136/136 | 113/113 | 103/112 | 182/186 | 183/185 | 154/156 |
| RPALLcl5 | TcId | 136/136 | 111/111 | 112/112 | 182/186 | 182/183 | 154/156 |

| **Clone** | **10101 TC VIC** | **10187 GA TET** | **10187 TA VIC** | **10187 TTA PET** | **7093 TC** | **8741 TA VIC** |
| --- | --- | --- | --- | --- | --- | --- |
| FCHcl1 | 104/104 | 111/113 | 131/131 | 175/175 | 160/160 | 194/196 |
| FCHcl2 | 104/104 | 111/113 | 131/131 | 175/175 | 160/160 | 194/196 |
| FCHcl3 | 104/104 | 111/113 | 131/131 | 175/175 | 160/160 | 194/196 |
| FCHcl4 | 104/104 | 111/113 | 131/131 | 175/175 | 160/160 | 194/196 |
| FCHcl5 | 104/104 | 111/113 | 131/131 | 175/175 | 160/160 | 194/196 |
| S.Antoniocl1 | 104/104 | 111/113 | 131/131 | 175/175 | 160/160 | 194/196 |
| S.Antoniocl2 | 104/104 | 111/113 | 131/131 | 175/175 | 160/160 | 194/196 |
| S.Antoniocl3 | 104/104 | 111/113 | 131/131 | 175/175 | 160/160 | 194/196 |
| S.Antoniocl4 | 104/104 | 111/113 | 131/131 | 175/175 | 160/160 | 194/196 |
| S.Antoniocl5 | 104/104 | 111/113 | 131/131 | 175/175 | 160/160 | 194/196 |
| LERcl11 | 104/104 | 111/113 | 131/131 | 175/175 | 160/160 | 194/196 |
| LERcl12 | 104/104 | 111/113 | 131/131 | 175/175 | 160/160 | 194/196 |
| LERcl14 | 104/104 | 111/113 | 131/131 | 175/175 | 160/160 | 194/196 |
| LERcl15 | 104/104 | 111/113 | 131/131 | 175/175 | 160/160 | 194/196 |
| LERcl16 | 104/104 | 111/113 | 131/131 | 175/175 | 160/160 | 194/196 |
| EHcl1 | 104/104 | 111/113 | 131/131 | 175/175 | 160/160 | 194/196 |
| EHcl2 | 104/104 | 111/113 | 131/131 | 175/175 | 160/160 | 194/196 |
| EHcl3 | 104/104 | 111/113 | 131/131 | 175/175 | 160/160 | 194/196 |
| EHcl4 | 104/104 | 111/113 | 131/131 | 175/175 | 160/160 | 194/196 |
| EHcl5 | 104/104 | 111/113 | 131/131 | 175/175 | 160/160 | 194/196 |
| XCHcl11 | 104/104 | 111/113 | 131/131 | 175/175 | 160/160 | 194/196 |
| XCHcl12 | 104/104 | 111/113 | 131/131 | 175/175 | 160/160 | 194/196 |
| XCHcl13 | 104/104 | 111/113 | 131/131 | 175/175 | 160/160 | 194/196 |
| XCHcl14 | 104/104 | 111/113 | 131/131 | 175/175 | 160/160 | 194/196 |
| XCHcl15 | 104/104 | 111/113 | 131/131 | 175/175 | 160/160 | 194/196 |
| NCHcl1 | 104/104 | 111/113 | 131/131 | 175/175 | 160/160 | 194/196 |
| NCHcl2 | 104/104 | 111/113 | 131/131 | 175/175 | 160/160 | 194/196 |
| NCHcl3 | 104/104 | 111/113 | 131/131 | 175/175 | 160/160 | 194/196 |
| NCHcl4 | 104/104 | 111/113 | 131/131 | 175/175 | 160/160 | 194/196 |
| NCHcl5 | 104/104 | 111/113 | 131/131 | 175/175 | 160/160 | 194/196 |
| LJVPcl6 | 104/104 | 111/113 | 131/131 | 175/175 | 160/160 | 194/196 |
| LJVPcl7 | 104/104 | 111/113 | 131/131 | 175/175 | 160/160 | 194/196 |
| LJVPcl8 | 104/104 | 111/113 | 131/131 | 175/175 | 160/160 | 194/196 |
| LJVPcl9 | 104/104 | 111/113 | 131/131 | 175/175 | 160/160 | 194/196 |
| LJVPcl10 | 104/104 | 111/113 | 131/131 | 175/175 | 160/160 | 194/196 |
| SMAcl3 | 104/104 | 111/113 | 131/131 | 175/175 | 160/160 | 194/196 |
| SMAcl7 | 104/104 | 111/113 | 131/131 | 175/175 | 160/160 | 194/196 |
| SMAcl8 | 104/104 | 111/113 | 131/131 | 175/175 | 160/160 | 194/196 |
| SMAcl9 | 104/104 | 111/113 | 131/131 | 175/175 | 160/160 | 194/196 |
| SMAcl10 | 104/104 | 111/113 | 131/131 | 175/175 | 160/160 | 194/196 |
| GCcl1 | 104/104 | 111/113 | 131/131 | 175/175 | 160/160 | 194/196 |
| GCcl2 | 104/104 | 111/113 | 131/131 | 175/175 | 160/160 | 194/196 |
| GCcl3 | 104/104 | 111/113 | 131/131 | 175/175 | 160/160 | 194/196 |
| GCcl4 | 104/104 | 111/113 | 131/131 | 175/175 | 160/160 | 194/196 |
| GCcl5 | 104/104 | 111/113 | 131/131 | 175/175 | 160/160 | 194/196 |
| DScl1 | 104/104 | 111/113 | 131/131 | 175/175 | 160/160 | 194/196 |
| DScl2 | 104/104 | 111/113 | 131/131 | 175/175 | 160/160 | 194/196 |
| DScl3 | 104/104 | 111/113 | 131/131 | 175/175 | 160/160 | 194/196 |
| DScl4 | 104/104 | 111/113 | 131/131 | 175/175 | 160/160 | 194/196 |
| DScl5 | 104/104 | 111/113 | 131/131 | 175/175 | 160/160 | 194/196 |
| RPALLcl1 | 104/104 | 111/113 | 131/131 | 175/175 | 160/160 | 194/196 |
| RPALLcl2 | 104/104 | 111/113 | 131/131 | 175/175 | 160/160 | 194/196 |
| RPALLcl3 | 104/104 | 111/113 | 131/131 | 175/175 | 160/160 | 194/196 |
| RPALLcl4 | 104/104 | 111/113 | 131/131 | 175/175 | 160/160 | 194/196 |
| RPALLcl5 | 104/104 | 111/113 | 131/131 | 175/175 | 160/160 | 194/196 |
